# Numerical study and orthogonal analysis of optimal performance parameters for vertical cooling of sintered ore

**DOI:** 10.1038/s41598-024-52583-6

**Published:** 2024-02-28

**Authors:** Weishu Wang, Shuailong Li, Juan Zhen, Jiawei Guo, Weihui Xu

**Affiliations:** 1 College of Energy and Power Engineering, North China University of Water Resources and Electric Power, Zhengzhou, 450045 China; 2 CSCEC SCIMEE Sci.& Tech. Co., Ltd, Chengdu, China

**Keywords:** Engineering, Mechanical engineering, Energy science and technology

## Abstract

The sinter cooler, essential for cooling hot sintered ore to a specific temperature, has seen recent advancements with the introduction of a vertical sinter cooling furnace. This innovation aims to enhance energy efficiency, reduce emissions, and improve waste heat recovery. Despite significant research, a quantitative analysis of factors impacting its cooling and heat transfer efficiency is lacking. This study utilizes the Euler model and local non-equilibrium thermodynamic theory to identify key factors affecting the gas–solid cooperative cooling process in the vertical cooler. Through an orthogonal experimental approach, the paper determines the optimal structural and operational parameters for the furnace. Key findings include that a gas–solid ratio of 1200m^3/t, inlet air temperature of 50 ℃, cooling section height of 6m, and diameter of 13.25m maximize efficiency, achieving a weighted range normalization value of 0.962. This configuration meets sintered ore cooling requirements while optimizing waste heat recovery. The study reveals that the impact on heat transfer efficiency is influenced primarily by the gas–solid ratio, followed by the cooling section's height, diameter, and inlet air temperature. These insights are crucial for enhancing the vertical sinter cooler's design, contributing to more energy-efficient and environmentally friendly sintering processes.

## Introduction

The steel industry is a significant basic industry, characterized as a long-term and high-energy consumption industry with an energy utilization rate of only 30–50%. Notably, the waste heat recovery rate from sintering is a mere 22%, leaving over 70% of waste heat unused^1^. Efficient industrial waste energy recovery is essential to achieve carbon peaking and carbon neutrality goals. The design of the vertical sinter cooling machine structure is based on the principle of dry quenching technology^2,3^. This machine employs closed-loop cooling gas circulation and gas–solid counterflow heat exchange technology, which fundamentally resolves the problems of air leakage and environmental pollution common in traditional cooling processes^4^, This innovation significantly enhances the energy recovery rate of high-temperature sinter and the heat quality of cooling gas^5^.

Vertical furnace cooling is a complex process and researchers have conducted numerous studies on the gas–solid heat transfer laws in vertical cooling furnaces. Li et al.^6^ utilized Comsol numerical simulation software to construct a mathematical model of gas–solid heat transfer within the waste heat tank, successfully simulating the cold air and sinter ore temperature distribution within the tank's ore layer, and revealing the influence of ore layer height and gas-to-material ratio on the gas–solid heat transfer process within the waste heat tank. Liu et al.^7^ employed the finite difference method to delve into the gas–solid heat exchange process within the sinter ore waste heat tank, focusing on analyzing the significant effects of gas-to-material ratio, cold air inlet temperature, and cooling air volume on gas–solid heat transfer within the tank. Guo et al.^8^ utilized CFD numerical simulation software to establish a two-dimensional gas–solid heat exchange model within the sinter ore waste heat tank, exploring the impact of cold air inlet temperature and cooling air volume on gas–solid heat exchange within the tank. Wang et al.^9^ obtained heat transfer related data for the waste heat tank's ore layer through experiments, derived a correlational formula for the heat transfer coefficient of the ore layer, and studied the relationships between cooling air volume, sinter ore particle diameter, ore layer height, and the heat transfer coefficient of the sinter ore layer. Li et al.^10^, integrating experimental data, further refined the formula for the heat transfer coefficient of the waste heat tank's ore layer. Feng et al.^11,12^ set up an experimental platform for gas–solid heat transfer in the sinter ore waste heat tank, fitting a correlational formula for gas–solid heat transfer in the ore layer based on experimental data, and examining the changes in gas–solid heat transfer coefficient with cooling air volume and sinter ore particle diameter. Huang et al.^13^, based on the actual operation of a sinter plant, studied the gas–solid convective heat transfer characteristics within the waste heat tank, identifying cooling air volume and cold air inlet temperature as key factors, and derived a correlational formula for gas–solid heat transfer in the ore layer. Through further in-depth research, Dong et al.^14,15^ introduced the concept of fire-use transfer coefficient, thereby deriving a formula for the fire-use transfer coefficient of the ore layer within the waste heat tank; they also fitted a correlational formula for the fire-use transfer coefficient of the sinter ore layer using experimental data. Building upon previous research results on gas–solid heat transfer within the sinter ore waste heat tank, Feng et al.^16,17^ primarily applied the theory of porous media and the theory of local non-thermal equilibrium to numerically simulate and analyze three-dimensional steady-state gas–solid heat transfer within the tank. The crux of this simulation was the integration of relevant experimental data, such as the inertial resistance coefficient of cold air flow, viscous resistance coefficient, gas–solid physical parameters, gas–solid heat transfer coefficients, into the mathematical model through User-Defined Functions (UDF) for simulation studies. Lastly, they analyzed the relationship between sinter ore inlet temperature, particle diameter, gas-to-material ratio, cold air inlet temperature, and gas–solid heat transfer within the waste heat tank. Shen et al.^18^, using synergy theory, studied the effects of gas-to-material ratio, ore layer height, and ore layer diameter on the synergy number of the sinter ore cooling process within the waste heat tank, suggesting a transition from a "tall and slim" to a "short and stout" tank design, emphasizing that gas-to-material ratio and ore layer height are the primary influencing factors, while the impact of ore layer diameter is relatively minor. Zhang^19^ analyzed the influence of gas-to-material ratio and bed height on gas–solid heat transfer during the vertical cooling process by using a mathematical model of porous media heat transfer. Tian^20^ conducted research on the optimization of operating parameters for the sintering cooler by establishing a three-dimensional flow and heat transfer calculation model, and established an analysis model^21^ with the recovery rate of dust as the objective function to analyze and optimize the optimal operating parameters and structural parameters for sinter cooling.

Although these studies have conducted a comprehensive exploration of the heat transfer laws in vertical sintering coolers, providing valuable insights for the optimization and design of these coolers, there is still a lack of in-depth quantitative analysis and comparison of the structural and operational parameters specific to vertical sintering coolers. The existing research on optimizing operational parameters primarily focuses on sinter annular coolers, which has limited applicability to vertical sintering cooling furnaces. Therefore, a comprehensive consideration of the cooling performance and energy-saving effects of vertical sintering coolers is essential. Conducting a quantitative analysis of their design parameters to determine the optimal structural and operational parameters is of great significance for the optimized design of vertical sintering coolers.

This paper, through the construction of a CFD model and numerical simulations, conducts an orthogonal analysis on the key factors affecting the cooling performance and energy-saving effects of vertical sintering coolers, including gas–solid ratio, inlet gas temperature, height, and diameter of the cooling section. The study quantifies the impact of structural and operational parameters on performance indicators. Furthermore, by employing a weighted comprehensive scoring method, the paper successfully identifies the optimal design parameters that enhance the comprehensive cooling performance and waste heat recovery efficiency of vertical sintering coolers.

## Model

### Mathematical models

The heat transfer process in a cooling furnace is a complex phenomenon, involving conduction, convection, and radiation. Accurately calculating the heat transfer process while considering all these factors is a challenging task that requires mathematical modeling and simulation experiments. The model assumes that: (1) the heat transfer process is steady-state and the furnace parameters remain constant; (2) the cooling air is treated as a compressible fluid, blower power remains constant; (3) the distribution of materials inside the furnace is considered to be an isotropic porous medium, and the sintered ore shape remains unchanged; (4) the heat transfer between the vertical furnace wall and the environment is ignored.

(1) Continuity equation:

1 $$\begin{array}{c}u\frac{\partial \left(\varepsilon {\rho }_{{\text{f}}}u\right)}{\partial x}+v\frac{\partial \left(\varepsilon {\rho }_{{\text{f}}}v\right)}{\partial y}+w\frac{\partial \left(\varepsilon {\rho }_{{\text{f}}}w\right)}{\partial z}=0\end{array}$$

where ε is the porosity of the mineral; ${\rho }_{{\text{f}}}$ is the density of air, kg/m^3^; $\mu$ is the apparent velocity of air, m/s; *u*, *v* and *w* are the partial velocities of air in the *x*, *y*, *z* directions respectively, m/s.

(2) Equations of motion:

The equation of motion is also known as the conservation of momentum equation, the Navier–Stokes (N-S) equation, and its expression is,

2 $$\begin{array}{c}\begin{array}{c}\frac{\partial }{\partial {x}_{j}}\left({u}_{i}{u}_{j}\right)=\left[\begin{array}{c}\frac{\partial }{\partial {x}_{j}}\left(\mu \frac{\partial {u}_{i}}{\partial {x}_{j}}\right)-\frac{\partial P}{\partial {x}_{j}}+\\ \frac{\partial }{\partial {x}_{j}}\left(\mu \frac{\partial {u}_{j}}{\partial {x}_{i}}\right)-\frac{2}{3}\frac{\partial }{\partial {x}_{i}}\left(\mu \frac{\partial {u}_{j}}{\partial {x}_{j}}\right)\end{array}\right]\\ +{g}_{i}-{f}_{i}+{S}_{i}\end{array}\end{array}$$

where ${u}_{j}$ is the apparent velocity of air in the *j* direction, m/s; ${g}_{i}$ is the volume force of air in the *i* direction, N/m^3^; ${f}_{i}$ is the resistance acting on a unit volume of air in the opposite direction, N/m^3^; *P* is the surface force of air, N/m^2^; ${S}_{i}$ is the source term in the *i* direction, N/m^3^.

When a fluid flows within a porous medium, the flow state is influenced by the porous medium, so the source term ${S}_{i}$ is added to Eq. (2), The expression for ${S}_{i}$ is:

3 $$\begin{array}{c}{S}_{i}=-\left(\frac{\mu }{\alpha }{u}_{i}+\frac{1}{2}{C}_{2}\rho \left|u\right|{u}_{i}\right)\end{array}$$

where $\frac{1}{\alpha }=\frac{150}{{D}_{p}^{2}}\frac{(1-\varepsilon )}{{\varepsilon }^{3}}$ is the coefficient of viscous resistance; ${C}_{2}=\frac{3.5}{{D}_{p}}\frac{(1-\varepsilon )}{{\varepsilon }^{3}}$ is the coefficient of inertial resistance.

The Ergun equation is used to calculate the coefficient of viscous drag and the coefficient of inertia of porous media:

4 $$\begin{array}{c}\frac{\Delta P}{L}=150\frac{(1-\varepsilon {)}^{2}}{{\varepsilon }^{3}}\frac{\mu {u}_{d}}{{d}_{p}^{2}}+1.75\frac{1-\varepsilon }{{\varepsilon }^{3}}\frac{\rho {u}_{d}^{2}}{{d}_{p}}\end{array}$$

where ${u}_{d}$ is the apparent flow rate; ${d}_{p}$ is the sinter particle size.

(3) Energy conservation equation.

The equation of fluid energy is:

5 $$\begin{array}{c}\varepsilon (\rho c{)}_{{\text{f}}}\frac{\partial {T}_{{\text{f}}}}{\partial \tau }+{\left(pc\right)}_{{\text{f}}}{u}_{{\text{f}}}\cdot \nabla {T}_{{\text{f}}}=\varepsilon \nabla \cdot \left({\lambda }_{{\text{f}}}\nabla {T}_{{\text{f}}}\right)+\varepsilon {q}_{{\text{f}}}+{h}_{\nu }\left({T}_{{\text{s}}}-{T}_{{\text{f}}}\right)\end{array}$$

The equation of solid energy is:

6 $$\begin{array}{c}\left(1-\varepsilon \right)(\rho c{)}_{{\text{s}}}\frac{\partial {T}_{{\text{s}}}}{\partial \tau }=\left(1-\varepsilon \right)\nabla \cdot \left({\lambda }_{{\text{s}}}\nabla {T}_{{\text{s}}}\right)+\left(1-\varepsilon \right){q}_{{\text{s}}}-{h}_{\upnu }\left({T}_{{\text{s}}}-{T}_{{\text{f}}}\right)\end{array}$$

where $(\rho c{)}_{f}$ is the volumetric heat capacity of the cooling air, J/(kg⋅K); $(\rho c{)}_{s}$ is the volumetric heat capacity of the sinter, J/(kg⋅K); ${T}_{f}$ is the cooling air temperature, K; ${T}_{{\text{s}}}$ is the sinter temperature, K; ${h}_{v}$ is the integrated volume heat transfer coefficient, W/(m^3^∙K); ${u}_{{\text{f}}}$ is the apparent speed of the cooling air, m/s; ${\lambda }_{{\text{f}}}$ is the thermal conductivity of the cooling air, W/(m⋅K); ${\lambda }_{{\text{s}}}$ is the thermal conductivity of the ore, W/(m⋅K); ${q}_{{\text{f}}}$ is the heat source term of the cooling air, W/m^3^; ${q}_{{\text{s}}}$ is the heat source term of the sinter, W/m^3^;

The expression for the combined heat transfer coefficient is:

7 $$\begin{array}{c}{h}_{v}=\frac{{\text{ln}}\frac{\left({T}_{s,\text{ in }}-{T}_{f,\text{ out }}\right)}{\left({T}_{s,\text{ out }}-{T}_{f,\text{ in }}\right)}}{\left(\frac{1}{{c}_{s}{m}_{s}}-\frac{1}{{c}_{f}{m}_{f}}\right)V}\end{array}$$

where ${T}_{s,\text{ in}}$ is the sinter inlet temperature, ${T}_{s,\text{ out}}$ is the sinter outlet temperature, ${T}_{f,\text{ in}}$ is the cooling air inlet temperature, ${T}_{f,\text{ out}}$ is the cooling air outlet temperature, K.

### Geometric model

**Figure 1:**
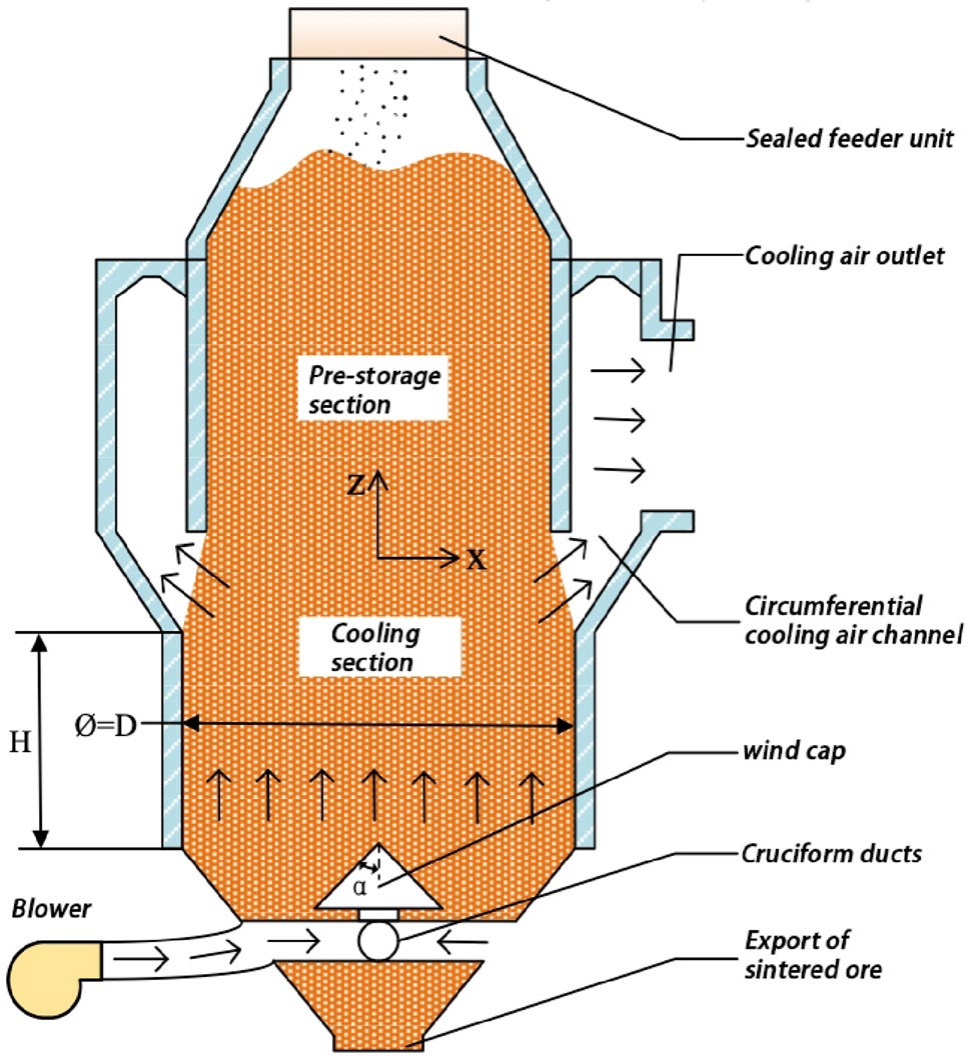
Schematic diagram of geometric structure of vertical sinter cooling furnace.

The vertical sinter cooling furnace unit receives cooling air from the air supply unit, which flows through the cross-shaped duct before entering the furnace through the air cap. Once the heat transfer process is complete, the air passes through the annular duct and exits through the outlet into the waste heat boiler for power generation. The fluid area of the cooling furnace structure is modeled for calculation purposes, with the geometric model illustrated in Fig. 1. The figure shows a section of the sinter cooling unit geometry at the central axis, where H represents the height of the cooling section, D represents the diameter of the cooling section, and α represents the angle of inclination of the wind cap.

### Mesh model

**Figure 2:**
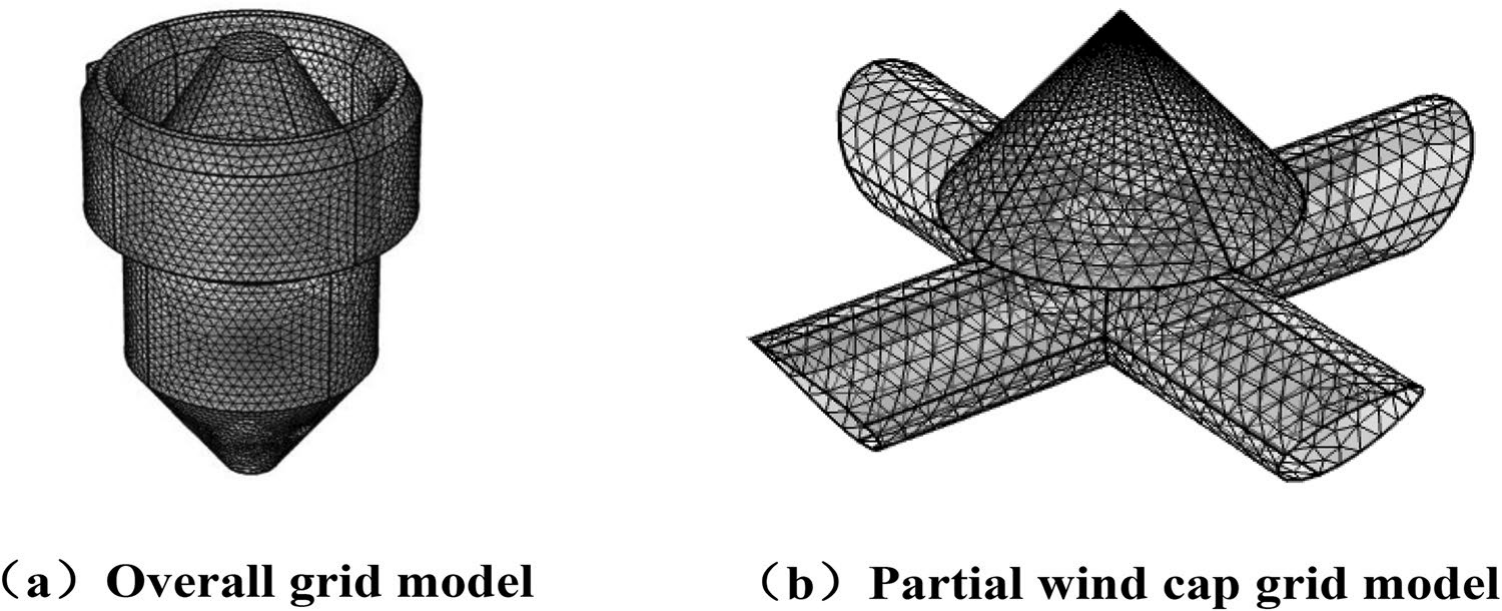
Grid model diagram of vertical sinter cooling furnace.

According to the geometric model based on Fluent Meshing software the model calculation area is discretized and an unstructured mesh is selected to mesh the overall model, the meshing model is shown in Fig. 2.

In one part, the local mesh of the wind cap was encrypted because the tip part of the cone is prone to mesh distortion. Mesh-independent verification was carried out, using five different mesh cell size control schemes, as shown in Table 1.

**Table 1:** Size control scheme of grid element.

Programme number	Model	Maximum grid cell size	Minimum grid cell size	Maximum grid cell growth rate	Curvature factor	Narrow area resolution
Grid 1	Overall	1.49	0.446	1.2	0.7	0.6
	Wind cap	1.83	0.229	1.45	0.5	0.6
Grid 2	Overall	0.996	0.297	1.15	0.6	0.7
	Wind cap	1.26	0.0914	1.4	0.4	0.7
Grid 3	Overall	0.788	0.149	1.13	0.5	0.8
	Wind cap	0.8	0.0343	1.35	0.3	0.85
Grid 4	Overall	0.55	0.0595	1.1	0.4	0.9
	Wind cap	0.457	0.0046	1.3	0.2	1
Grid 5	Overall	0.342	0.0223	1.08	0.3	0.95
	Wind cap	0.263	0.0032	1.2	0.2	1

The flow of cooling air inside the furnace is assumed to be incompressible, and the boundary conditions of the grid model are established as follows: (1) The flow of cooling air in the air duct is ignored, and the upper surface of the cone at the air cap is set as the inlet for cooling air velocity. (2) The cooling air outlet is designated as the pressure outlet, with a static pressure of 0 at the outlet. (3) The sintered ore inlet serves as the flow inlet, and the flow size is set to 102 kg/s, while the bottom is set as the pressure outlet. (4) The wall surface is considered as an adiabatic wall surface. (5) Sintered ore is only present in the interior of the furnace, so the region of the grid model other than the annular duct is set as a porous media area. The porous area is homogenized with a porosity of 0.4 and a particle size of 11.2 mm^22,23^.

Due to the impact of the number and quality of model grids on the results of simulation calculations, a grid independence verification is conducted on the mesh model prior to numerical computation. Based on the grid size schemes presented in Table 1, grid independence verification is carried out for the operating conditions shown in Table 2 to simulate the heat transfer process in the vertical sinter cooling furnace.

**Table 2:** Simulated working parameters.

Parameter	Numerical
Gas–solid ratio	1000m^3^/t
Sinter ore inlet temperature	1023.15K
Inlet air temperature	313.5 K
Height of cooling section	7 m
Cooling section diameter	11.25 m
Inlet air angle	40°

**Figure 3:**
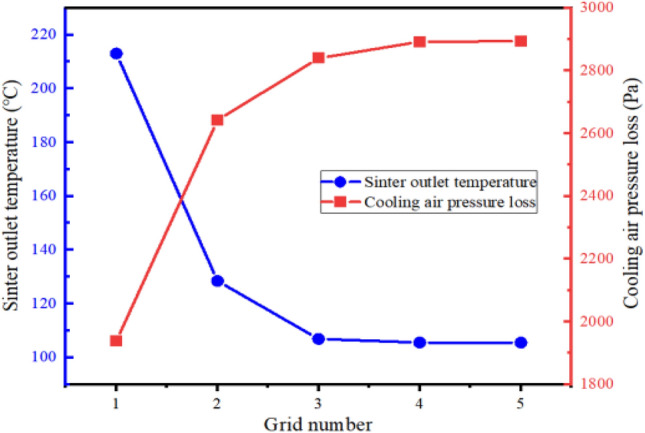
Size control scheme of grid element.

The sinter ore outlet temperature and cooling air pressure loss are taken as the criterion for calculation accuracy, and the sinter ore outlet temperature and cooling air pressure loss under different scenarios are shown in Fig. 3.

The figure presented in this section indicates significant differences between Grid 1, Grid 2, and the encrypted calculation results, thereby failing to meet the required accuracy level. Although Grid 3 demonstrated comparatively less variation, there was still some difference. Grid 5 displayed no significant variation in the calculation results despite reducing the size and increasing the density compared to Grid 4. Therefore, after considering all factors, Grid 4 was selected for meshing.

Conduct a reliability verification of the model. The vertical cooling process of sintered ore has not yet been widely applied in existing industrial practices. Therefore, the paper chose to compare the heat transfer calculation model’s accuracy with experimental data of single-grade sintered ore from the study by Zhang^1^. The operating parameters are as follows: gas velocities of 0.4 m/s, 0.8 m/s, 1.2 m/s, 1.6 m/s, and 2.0 m/s, particle equivalent diameter of 11.45 mm, and cooling section height of 0.455 m. By analyzing the trend of gas flow resistance per unit height /L with the variation of cooling air inlet velocity under the aforementioned operating parameters, the accuracy of the established model can be assessed. The comparative results are shown in Table 3. Comparative analysis between the numerical values obtained from the simulation and the actual experimental values reveals that the error in the resistance per unit height of the material layer is within 10%, which meets the requirements for model validation. This indicates that the numerical simulation established in the paper is reliable.

**Table 3:** Comparative results.

Air velocity (m/s)	Resistance loss per unit mineral layer height (Pa)		
	Experimental value (Pa/m)	Calculated value (Pa/m)	Relative error (%)
0.4	318.17	315.33	0.89
0.8	961.25	958.45	0.29
1.2	1857.55	1699.65	8.50
1.6	3105.14	3396.75	9.39
2.0	4556.43	4383.99	3.78

### Analysis of typical working conditions

**Figure 4:**
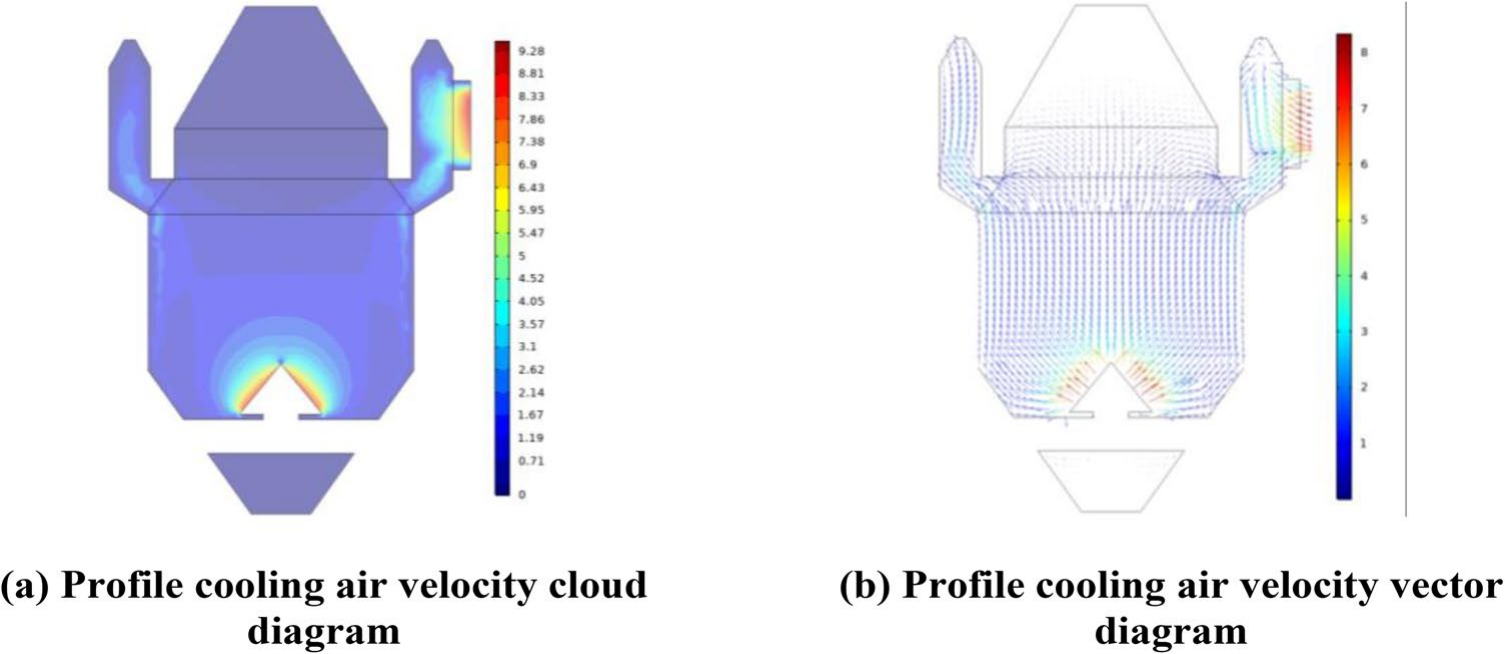
Profile velocity distribution.

The heat transfer effect of the sinter vertical cooling furnace is influenced by the gas–solid ratio and the inlet air temperature as main operating parameters, and the height and diameter of the cooling section, as well as the inlet air angle of the cooling air, as main structural parameters. Figure 4 presents the velocity cloud and velocity vector diagram along the furnace heat transfer process profile. The diagram indicates that the cooling air is blown into the furnace from the wind cap in a direction perpendicular to the wind cap. At this point, the velocity of the cooling air is at its maximum and tends to become uniform as the height of the cooling section increases. The porous media area's small particle size and porosity cause more resistance within the cooling section, causing the cooling air velocity to rapidly decrease. Once the cooling air enters the annular duct, the air pressure outside is lower than the internal air pressure, and the absence of sintered ore material in the duct increases the cooling air velocity. Additionally, the air velocity increases closer to the outlet.

**Figure 5:**
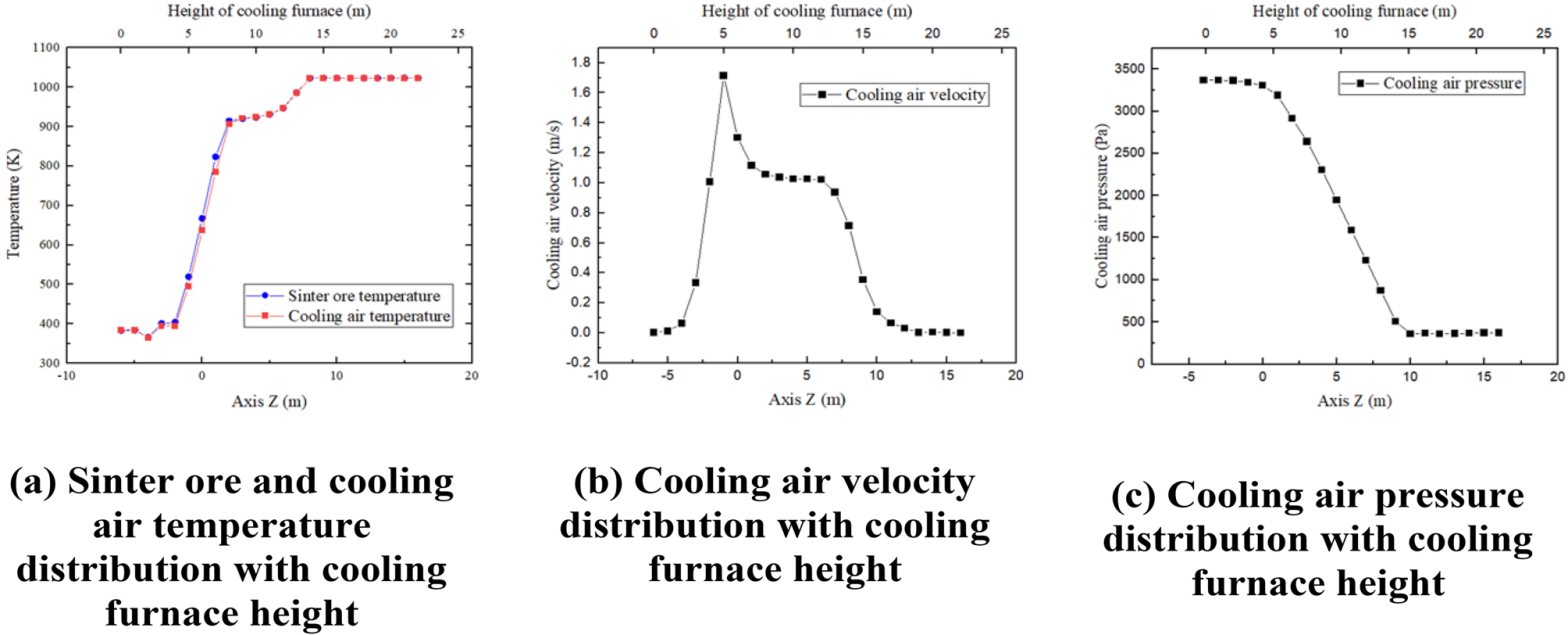
Temperature, velocity and pressure distribution along the height direction in the cooling furnace.

To study the distribution of parameters along the axial direction in the furnace, we took 23 cross-sections of the cooling furnace in the z-direction and calculated the average parameters for each cross-section. The obtained results are presented in Fig. 5.

As can be seen in Fig. 5b, from the bottom to z = − 2 m, the cooling air velocity is close to stagnation, and the temperature of sintered ore and cooling air in this section is basically unchanged and remains the same as the temperature of the falling material. From the bottom to z = 2 m, the cooling air velocity is close to stagnation, and the temperature of sinter ore and cooling air in this part is basically unchanged and remains the same as the drop temperature. − 2 m to 2 m is the main heat exchange area between sinter ore and cooling air, the cooling air velocity is maximum, and the cooling air and sinter ore are in partial non-thermal equilibrium. 2 m to 7 m section is the secondary cooling area in the cooling furnace, the cooling air velocity remains stable, and the sinter ore and cooling air are basically in thermal equilibrium, with the height increasing, the heat exchange effect is partially improved. 7 m and above is the pre-storage section of the vertical sinter cooling furnace, and the temperature of sinter ore in this section hardly decreases. From Fig. 5c it can be seen that the variation of the cooling air pressure is influenced by the resistance in the porous media area and decreases uniformly from the wind cap to the entrance of the annular duct, the pre-storage section has a lower pressure due to the large outflow of air at the annular duct.

## Orthogonal experimental

There are many factors affecting the heat transfer and resistance characteristics of sinter ore, and a comprehensive test considering the influence of all factors on the heat transfer characteristics of the cooling furnace would make the test extremely large and redundant. An orthogonal test design allows the consideration of many different factors at the same time and reduces the workload significantly, thus enabling the optimum design of parameters for a vertical sinter cooling furnace.

### Orthogonal table design

The main factors influencing the heat transfer effect and resistance characteristics of the vertical sinter cooling furnace are: gas—solid ratio, inlet air temperature, cooling section height and cooling section diameter. Therefore, a 4-factor orthogonal table is used, as shown in Table 4.

**Table 4:** Factor level table.

Parameters	Gas–solid ratio (m^3^/t)	Inlet air temperature (℃)	Height of cooling section (m)	Diameter of cooling section (m)
1 level	900	20	6	10.25
2 level	1000	30	7	11.25
3 level	1100	40	8	12.25
4 level	1200	50	9	13.25

The gas–solid ratio has a large impact on the heat transfer effect of the cooling furnace, taking 900–1200. For the inlet air temperature, considering 20 ℃ as the standard environmental temperature, so take 20 ℃ as the benchmark, and select representative working conditions 20–50 ℃. The height of the cooling section with a small change has a small effect on the cooling effect, expand the height condition interval, the condition interval is taken as 1 m, and take 6–9 m condition for calculation. The diameter of the cooling section is similar to the height of the cooling section, the working condition interval is taken as 1 m, and the diameter of the cooling section is taken as 10.25–13.25 m.

As can be seen from Table 4, the test contains a total of 4 levels and 4 factors. Therefore, a orthogonal table was used to design the test conditions. The orthogonal test table is shown in Table 5.

**Table 5:** Orthogonal test table.

Test number	Parameter 1	Parameter 2	Parameter 3	Parameter 4
	Gas–solid ratio (m^3^/t)	Inlet air temperature (℃)	Height of cooling section (m)	Diameter of cooling section (m)
1	900	20	6	10.25
2	900	30	7	11.25
3	900	40	8	12.25
4	900	50	9	13.25
5	1000	20	7	12.25
6	1000	30	6	13.25
7	1000	40	9	10.25
8	1000	50	8	11.25
9	1100	20	8	13.25
10	1100	30	9	12.25
11	1100	40	6	11.25
12	1100	50	7	10.25
13	1200	20	9	11.25
14	1200	30	8	10.25
15	1200	40	7	13.25
16	1200	50	6	12.25

### Data quantification

The value of the exergy carried by the cooling air represents the amount of energy it can utilise. The meaning of the exergy efficiency represents the ability of the cooling air to convert energy from the hot sintered ore material. In engineering, where the difference in exergy efficiency is small, it is generally more important to focus on the energy that can be carried at the exit of the cooling air.

The exergy value carried by the cooling air represents the work available for waste heat recovery and should have a larger value, which is a positive indicator. The expressions are as follows:

8 $$\begin{array}{c}{E}_{{T}_{i}}=m{c}_{P}\left({T}_{i}-{T}_{o}\right)\left[1-\frac{{T}_{o}}{{T}_{i}-{T}_{o}}{\text{ln}}\frac{{T}_{i}}{{T}_{o}}\right]\end{array}$$

where $m$ mass flow rate, kg/s; ${c}_{P}$ specific heat, J/(kg∙K); ${T}_{i}$ average temperature, K; ${T}_{o}$ environmental temperature, K; ${E}_{{T}_{i}}$ the energy amount of cooling air at temperature, J.

In addition to this, the material layer resistance loss represents the amount of work done by the blower, i.e. the energy consumption. Therefore, the value of the cooled air carried by the blower and the material layer resistance loss were chosen as the evaluation indicators for the test.

Quantifying the data, the maximum difference obtained from the test results and the calculated extreme difference are used to define a extreme differential value dimensionless number that converts multiple indicators into a single indicator for evaluation and enables an intuitive measurement of the test results.

In the weighted composite scoring method, the *i*th positive indicator is ${Y}_{i}$ and the ${\text{i}}$ th positive indicator in the $t$ th trial is noted as ${Y}_{ii}$.($1\le t\le n$, $n$ is the number of tests). The "polarized" value of the positive indicator ${Y}_{i}$ for the $t$ th test data is:

9 $$\begin{array}{c}{Y}_{ii}^{\mathrm{^{\prime}}}=\frac{{Y}_{ii}-{Y}_{i,{\text{mi}}n}}{{R}_{i}}=\frac{{Y}_{ii}-{Y}_{i,{\text{mi}}n}}{{Y}_{i,{\text{ma}}x}-{Y}_{i,{\text{mi}}n}}\end{array}$$

where ${Y}_{ii}^{\mathrm{^{\prime}}}$ is the extreme differential value of the positive indicator for the $t$ th test data; ${Y}_{i,min}$ is the minimum value of the positive indicator in the test data; ${R}_{i}$ is the maximum difference of the positive indicator ${Y}_{i}$ in the test data; ${Y}_{i,max}$ is the maximum value of the positive indicator ${Y}_{i}$ in the test data.

The material layer resistance loss is the amount of work done by the outside world during the heat exchange between the sintered ore and the cooling air, and in practical engineering, the smaller the value the better, so the material layer resistance loss is chosen as a negative indicator. In the weighted composite scoring method, the $j$ th negative indicator is ${Y}_{j}$, and the $j$ th negative indicator in the $t$ th test is noted as ${Y}_{tj}$. The "polarized" value of the negative indicator ${Y}_{j}$ in the tth test data is:

10 $$\begin{array}{c}{Y}_{tj}^{\mathrm{^{\prime}}}=\frac{{Y}_{j,max}-{Y}_{tj}}{{R}_{j}}=\frac{{Y}_{j,max}-{Y}_{tj}}{{Y}_{j,max}-{Y}_{j,min}}\end{array}$$

where ${Y}_{tj}^{\mathrm{^{\prime}}}$ is the extreme differential value of the negative indicator ${Y}_{j}$ for the $t$ th test data; ${Y}_{j,max}$ is the maximum value of the negative indicator ${Y}_{j}$ in the test data; ${R}_{j}$ is the maximum difference of the positive indicator ${Y}_{j}$ in the test data; ${Y}_{j,min}$ is the minimum value of the positive indicator ${Y}_{j}$ in the test data.

The evaluation indicators are quantified uniformly and the weight of each indicator is determined. The weight of the $i$ th positive indicator is denoted as ${W}_{i}$ and the weight of the $j$ th negative indicator is denoted as ${W}_{j}$. The weighting of each indicator should satisfy:

11 $$\begin{array}{c}{\sum }_{i=1}^{n} {W}_{i}+{\sum }_{j=1}^{m} {W}_{j}=1\end{array}$$

where $n$ is the total number of positive indicators; $m$ is the total number of negative indicators.

Unified indicator quantification values (weighted polarized values) is:

12 $$\begin{array}{c}{Y}_{t}=\sum {W}_{i}{Y}_{ti}^{\mathrm{^{\prime}}}+\sum {W}_{j}{Y}_{tj}^{\mathrm{^{\prime}}}\end{array}$$

${Y}_{t}$ converts the problem of optimal level combinations under multiple indicators into an optimal level combination for a single indicator.

## Results and discussion

### Data analysis

In equations, Numerical simulations were carried out and calculated for each of the test conditions in Table 5 and the results are shown in Table 6.

**Table 6:** Orthogonal test data table.

Test number	Sinter ore outlet temperature (℃)	Cooling air outlet temperature (℃)	exergy value carried by cooling air (kW)	Material layer resistance losses (Pa)
1	131.29	666.45	26,043.06	2764.58
2	144.79	643.53	25,732.94	2641.61
3	161.14	615.87	25,306.94	2500.65
4	174.17	585.95	24,954.59	2359.14
5	71.34	613.63	28,274.38	2389.10
6	89.35	613.50	28,357.47	1733.05
7	104.56	630.91	28,351.70	4501.07
8	119.32	627.60	28,234.98	3302.89
9	30.22	551.00	30,317.46	2463.77
10	44.81	561.13	30,360.50	3294.27
11	65.80	612.51	31,443.83	2660.47
12	84.72	616.39	31,393.56	3787.37
13	25.70	491.03	31,409.55	4022.45
14	42.65	518.26	32,142.86	4390.38
15	43.86	524.86	32,624.84	2288.70
16	58.17	557.83	33,427.71	2339.84

The test data combined with the extreme differential values of Eq. (8) were calculated according to Table 6 and the results are shown in Table 7, where The choice of weights ${W}_{1}=0.7$, ${W}_{2}=0.3$.

**Table 7:** Extreme difference of test results.

Test number	1	2	3	4	5	6	7	8
Exergy value ${Y}_{t1}^{\prime}/(Kw)$	0.128	0.092	0.042	0.000	0.392	0.402	0.401	0.387
Material layer resistance losses ${Y}_{t2}^{{{\prime}}}/Pa$	0.627	0.672	0.723	0.774	0.763	1.000	0.000	0.433
Weighted polarisation values ${Y}_{t}$	0.278	0.266	0.246	0.232	0.503	0.581	0.281	0.401

Test number	9	10	11	12	13	14	15	16
Exergy value ${Y}_{t1}^{\prime}/(Kw)$	0.633	0.638	0.766	0.760	0.762	0.848	0.905	1.000
Material layer resistance losses ${Y}_{t2}^{\mathrm{^{\prime}}}/Pa$	0.736	0.436	0.665	0.258	0.173	0.040	0.799	0.781
Weighted polarisation values ${Y}_{t}$	0.664	0.577	0.736	0.609	0.585	0.606	0.873	0.934

As can be seen from Table 7, Scheme 16 is the best working condition among the 16 groups of tests, when the gas–solid ratio is $1200{{\text{m}}}^{3}\text{/t}$, the inlet air temperature is 50 °C, the height of the cooling section is 6 m and the diameter of the cooling section is 12.25 m, the heat exchange economy between the sintered ore and the cooling air is the best among the 16 schemes.

### Determination of optimal parameters

The above analysis indicates the best of the test conditions, but there are only 16 test conditions and theoretically better conditions may exist beyond these 16, so the data needs to be analysed to determine the best combination of parameters.

The weighted polarisation values for each parameter at each level were averaged and calculated according to Table 3 and the results are shown in Table 8.

**Table 8:** Level table of weighted polarisation values.

Parameters	1	2	3	4
${I}_{i}$	0.256	0.508	0.632	0.443
$I{I}_{i}$	0.441	0.508	0.563	0.497
$II{I}_{i}$	0.647	0.534	0.479	0.565
$I{V}_{i}$	0.750	0.544	0.419	0.588
${R}_{i}$	0.494	0.037	0.213	0.144

In Table 8, ${I}_{i}$, $I{I}_{i}$, $II{I}_{i}$, $I{V}_{i}$ are the average of all weighted polarisation values for parameter $i$ at levels 1, 2, 3 and 4 respectively, and $1<i<4$. ${R}_{i}$ is the extreme difference under the same parameter and is the difference between the maximum weighted polarisation value and the minimum weighted polarisation value in parameter $i$.

By using Table 8, the theoretically optimal parameter combinations can be derived. For parameter 1, level 4 is the highest weighted polarisation value; in parameter 2, level 4 is the highest weighted polarisation value; in parameter 3, level 1 is the highest weighted polarisation value; in parameter 4, level 4 is the highest weighted polarisation value. Therefore, the theoretical optimum parameters are: gas–solid ratio of 1200 ${{\text{m}}}^{3}\text{/t}$, inlet air temperature of 50 °C, cooling section height of 6 m and cooling section diameter of 13.25 m.

The theoretically optimal parametric working conditions are simulated numerically and the weighted polarised values are calculated for the data according to the previous method. For comparison purposes, the values of ${R}_{i}$ in the 16 previously described solutions are still used in the calculations. The calculation equations are as follows:

13 $$\begin{array}{c}{Y}_{m}^{\prime}=\frac{0.7\left({Y}_{mi}-{Y}_{i,min}\right)}{{R}_{i}}+\frac{0.3\left({Y}_{j,max}-{Y}_{mj}\right)}{{R}_{j}}\end{array}$$

where ${Y}_{m}^{\prime}$ is the weighted extreme difference value of the optimal solution; ${Y}_{mi}$ is the value of the positive indicator of the optimal solution; ${Y}_{i,min}$ is the minimum value of the positive indicator among the 16 solutions; ${R}_{i}$ is the maximum differential value of the positive indicator among the 16 solutions; ${Y}_{j,max}$ is the maximum value of the negative indicator among the 16 solutions; ${Y}_{mj}$ is the value of the negative indicator of the optimal solution; ${R}_{j}$ is the maximum differential value of the negative indicator among the 16 solutions.

The results of the calculations are shown in Table 9. As can be seen from Table 8, the weighted combined value for the optimal parameters is 0.962, which is higher than the maximum value of 0.934 in the 16 scenarios. therefore, the optimal parameters are determined as a gas–solid ratio of 1200 ${{\text{m}}}^{3}\text{/t}$, an inlet air temperature of 50 °C, a cooling section height of 6 m and a cooling section diameter of 13.25 m.

**Table 9:** Calculation results of optimal parameters.

Sinter ore outlet temperature (℃)	Cooling air outlet temperature (℃)	Exergy value carried by cooling air (kW)	Material layer resistance losses (Pa)	Weighted combined value
55.702	547.64	33,292.11	1979.92	0.962

Further analysis shows that the horizontal extreme differences ${R}_{i}$ for the different parameters are parameter 1 > parameter 3 > parameter 4 > parameter 2. This value reflects the magnitude of the influence on the heat transfer between the sintered ore and the cooling air. Thus, among the factors influencing the cooling process in the vertical furnace, the gas-to-solid ratio, the height of the cooling section, the diameter of the cooling section and the inlet air temperature have a decreasing influence on the heat transfer effect in that order (Supplementary Data).

## Conclusion

In order to investigate the flow and heat exchange characteristics of high temperature sintered ore gas–solid counter-current cooling, the orthogonal test method was used to simulate and study the key influencing factors of the gas–solid cooperative cooling process of the cooling furnace within reasonable structural and operating parameters, and to optimise the parameters of the sintering furnace, and to determine the best structural and operating parameters, with the following main conclusions:

1. Close to the cooling air inlet, the temperature gradient is the greatest, where the heat exchange is most intense. After passing through the wind cap, the temperature tends to stabilize, approaching the temperature at the material discharge port. At the same location, the temperature of the cooling air is slightly lower than the temperature of the sintered ore.
2. The theoretical optimum parameters were determined by the weighted integrated scoring method: gas–solid ratio of 1200 ${{\text{m}}}^{3}\text{/t}$, inlet air temperature of 50 °C, cooling section height of 6 m and cooling section diameter of 13.25 m. The optimum parameters were verified by simulation and the polarisation value reached 0.962, which is higher than the optimum value among the 16 solutions, with the best waste heat recovery effect.
3. The level extreme difference values of operating and structural parameters reflect the size of the influence of heat exchange between sintered ore and cooling air. Among the influencing factors of the sintered ore vertical furnace cooling process, the gas–solid ratio, cooling section height, cooling section diameter and inlet air temperature have a decreasing degree of influence on the heat exchange effect in turn.

### Supplementary Information

Supplementary Information.

## Data Availability

The datasets used and/or analysed during the current study available from the corresponding author on reasonable request.
